# Standardized ileal digestibility of amino acids in soybean meal fed to non-pregnant and pregnant sows

**DOI:** 10.1186/s40104-023-00928-y

**Published:** 2023-10-06

**Authors:** Ke Wang, Ya Wang, Lei Guo, Yong Zhuo, Lun Hua, Lianqiang Che, Shengyu Xu, Ruinan Zhang, Jian Li, Bin Feng, Zhengfeng Fang, Xuemei Jiang, Yan Lin, De Wu

**Affiliations:** Institute of Animal Nutrition, Key Laboratory for Animal Disease-Resistance Nutrition of China, Ministry of Education, Sichuan Agricultural University, 211 Huimin Road, Wenjiang District, Chengdu, 611130 Sichuan China

**Keywords:** Amino acids, Sows, Soybean meal, Standardized ileal digestibility

## Abstract

**Background:**

Two studies were designed to determine standard ileal crude protein (CP) and amino acid (AA) digestibility of soybean meal (SBM) from different origins fed to non-pregnant and pregnant sows. Seven solvent-extracted SBMs from soybeans produced in the USA, Brazil, and China were selected. In Exp. 1, eight different diets were created: a nitrogen (N)-free diet and 7 experimental diets containing SBM from different origins as the only N source. Eight non-pregnant, multiparous sows were arranged in an 8 × 8 Latin square design (8 periods and 8 diets). In Exp. 2, the diet formula was the same as in Exp. 1. Eight gestating sows (parity 3) were assigned to 4 different diets in a replicated 4 × 3 Youden square design (three periods and four diets) in mid-gestation and again in late-gestation stages.

**Results:**

When fed to non-pregnant and late-gestating sows, the standardized ileal digestibility (SID) of CP and most AAs from different SBM were not significantly different (*P* > 0.05). When fed to mid-gestating sows, the SID values for Arg, His, Lys, Phe, Cys, Gly, Ser, and Tyr in SBM 1 were lower than in SBM 4 and 5 (*P* < 0.05), whereas SID for Leu from SBM 5 was higher than in SBM 1 and 4 (*P* < 0.05). SID values for Ile, Ala, and Asp from SBM 4 were lower than in SBM 1 and 5 (*P* < 0.05). Sows had significantly greater SID values for Lys, Ala, and Asp during mid-gestation when compared with late-gestation stages (*P* < 0.05). Mid-gestating sows had greater SID value for Val and lower SID value for Tyr when compared with non-pregnant and late-gestating sows (*P* < 0.01), whereas non-pregnant sows had significantly greater SID value for Met when compared with gestating sows (*P* < 0.01).

**Conclusions:**

When fed to mid-gestating sows, the SID values for most AAs varied among SBM samples. The SID values for Lys, Met, Val, Ala, Asp, and Tyr in SBM were affected by sow gestation stages. Our findings provide a cornerstone for accurate SBM use in sow diets.

## Background

Feed resource shortages have become serious bottlenecks restricting the development of sustainable animal husbandry. Feed costs can account for more than 60% of total swine production costs [1]. Globally, soybean meal (SBM) is the main amino acid (AA) source in swine diets because of its well-balanced AA composition [2]. Accurate assessment of SBM nutritional values for sows at different gestation stages is essential for efficient livestock production [3]. From previous reports, differences in digestible nutrients in SBM from different origins can affect their efficient application in growing pig feed [4, 5]. SBM produced in the USA contains more indispensable AAs than SBM from Argentina and China, and the standardized ileal digestibility (SID) levels for crude protein (CP) and most AAs in SBM from the USA are greater than in SBM from Brazil, India, and Argentina when fed to growing pigs [6].

However, SBM nutritive values may vary when fed to pigs at different physiological stages. We observed that the metabolizable energy and nitrogen (N) net utilization values of SBM were greater in late-gestating sows when compared with mid-gestating sows [3]. Additionally, the SIDs of CP and AAs in dehulled SBM were less in pigs with a body weight (BW) under 20 kg when compared with pigs over 20 kg [7]. When compared with growing pigs, pregnant sows had higher SID values of CP and all AAs, except for Asp and Trp, when fed SBM [8]. Because of the need to surgically modify sows, it is difficult to evaluate the SID values of AAs in feed ingredients in multiparous sows. Currently, when formulating diets for sows, the SID values of AAs in ingredients are mostly based on values from growing pigs [9, 10]. Thus, accurate SBM nutrient digestibility information at specific sow gestation stages is required if precise sow feed formulations are to be achieved.

Consequently, our objectives were to determine SID values for CP and AAs in SBM from different sources in pregnant and non-pregnant multiparous sows, and analyze the effects of different pregnancy stages on the ileal digestibility of AAs in SBM.

## Materials and methods

The Animal Care and Use Committee of Sichuan Agricultural University reviewed and approved all study protocols (SICAU20210038). Seven solvent-extracted SBM samples were collected from the main SBM-producing areas in China (Table 1). The soybeans were grown in the USA, Brazil, and China. SBM chemical composition values are shown in Tables 2 and 3.

**Table 1:** Soybean meal origins

**No.**	**Source of soybean**	**Type of soy**^**a**^	**Plants**^**b**^	**Location of plants in China**
SBM 1	China	Conventional	A1	Heilongjiang
SBM 2	China	Conventional	A2	Heilongjiang
SBM 3	Brazil	RR GM	B	Sichuan
SBM 4	Brazil	RR GM	C	Sichuan
SBM 5	US	RR GM	D	Hebei
SBM 6	US	RR GM	E	Tianjin
SBM 7	US	RR GM	F	Chongqing

^a^RR GM: Roundup-ready genetically modified

^b^The same capital letter indicates that beans were processed in the same plant while different numbers indicate different soybean varieties

**Table 2:** Nutrient composition (% dry matter, unless otherwise indicated) of different soybean meal

**Item**	**SBM 1**	**SBM 2**	**SBM 3**	**SBM 4**	**SBM 5**	**SBM 6**	**SBM 7**	**Mean**	**Maximum**	**Minimum**	**CV**
Dry matter	89.39	89.07	87.63	87.94	87.87	88.18	87.00	88.15	89.39	87.00	0.94
Gross energy, kcal/kg	4,423.11	4,477.45	4,518.51	4,534.24	4,517.13	4,535.27	4,590.31	4,513.72	4,590.31	4,423.11	1.16
Crude protein	50.07	48.62	50.13	53.42	49.24	48.50	50.02	50.00	53.42	48.50	3.31
Ether extract	2.79	0.97	1.15	1.01	1.49	2.14	1.11	1.52	2.79	0.97	45.38
Crude fiber	10.02	5.48	6.00	3.70	4.69	6.52	4.54	5.85	10.02	3.70	35.37
Neutral detergent fiber	15.44	9.79	9.71	6.66	9.91	11.37	8.41	10.18	15.44	6.66	26.91
Acid detergent fiber	10.99	6.99	6.60	4.56	6.32	8.69	5.29	7.06	10.99	4.56	30.72
Insoluble dietary fiber	22.15	20.43	22.37	17.06	21.40	21.77	19.77	20.71	22.37	17.06	8.97
Soluble dietary fiber	2.53	2.18	3.14	1.39	2.38	2.38	1.85	2.26	3.14	1.39	24.24
Total dietary fiber	24.72	22.57	25.56	18.42	23.79	24.16	21.61	22.98	25.56	18.42	10.45
Ash	8.31	6.95	6.58	6.74	6.45	6.21	6.90	6.88	8.31	6.21	9.92
Carbohydrates											
Maltose	0.45	0.36	0.21	0.19	0.19	0.14	0.29	0.26	0.45	0.14	42.39
Sucrose	4.03	3.59	3.42	7.73	4.55	2.72	8.62	4.95	8.62	2.72	46.18
Stachyose	1.64	1.87	1.59	2.05	1.16	1.63	2.01	1.71	2.05	1.16	17.89
Raffinose	0.34	0.46	0.79	0.94	0.70	0.78	0.70	0.67	0.94	0.34	30.59
Indispensable amino acids											
Arg	3.07	3.60	3.79	3.81	3.65	3.39	3.40	3.53	3.81	3.07	7.44
His	1.24	1.25	1.35	1.28	1.18	1.30	1.28	1.27	1.35	1.18	4.18
Ile	1.77	2.11	2.24	2.29	2.12	2.10	2.03	2.09	2.29	1.77	8.03
Leu	3.15	3.74	3.95	4.04	3.73	3.72	3.67	3.71	4.04	3.15	7.64
Lys	2.74	3.18	3.15	3.24	2.90	2.90	3.15	3.04	3.24	2.74	6.20
Met	0.74	0.79	0.83	0.83	0.84	0.82	0.81	0.81	0.84	0.74	4.25
Phe	2.02	2.43	2.59	2.64	2.41	2.43	2.39	2.42	2.64	2.02	8.25
Thr	1.69	1.94	2.00	2.08	1.91	1.87	1.89	1.91	2.08	1.69	6.35
Trp	0.55	0.67	0.65	0.69	0.61	0.52	0.63	0.62	0.69	0.52	10.11
Val	1.85	2.13	2.25	2.32	2.13	2.12	2.23	2.15	2.32	1.85	7.04
Dispensable amino acids											
Ala	1.85	2.05	2.13	2.21	2.05	2.04	2.28	2.09	2.28	1.85	6.64
Asp	4.69	5.51	5.67	5.92	5.42	5.36	5.37	5.42	5.92	4.69	6.98
Cys	0.93	1.02	1.05	1.11	1.04	1.01	1.03	1.03	1.11	0.93	5.23
Glu	7.37	9.00	9.40	9.84	8.91	8.91	8.85	8.90	9.84	7.37	8.57
Gly	1.80	2.03	2.10	2.16	2.03	1.98	2.09	2.03	2.16	1.80	5.72
Pro	2.07	2.43	2.60	2.65	2.46	2.45	2.15	2.40	2.65	2.07	9.01
Ser	2.14	2.51	2.57	2.68	2.47	2.44	2.38	2.46	2.68	2.14	6.90
Tyr	1.55	1.84	1.89	1.91	1.88	1.76	1.98	1.83	1.98	1.55	7.68

**Table 3:** Mineral and vitamin concentrations in different soybean meals (mg/kg dry matter, unless otherwise indicated)

**Item**	**SBM 1**	**SBM 2**	**SBM 3**	**SBM 4**	**SBM 5**	**SBM 6**	**SBM 7**	**Mean**	**Maximum**	**Minimum**	**CV**
Macro minerals											
Calcium, %	0.36	0.39	0.41	0.43	0.39	0.44	0.40	0.40	0.44	0.36	6.68
Phosphorus, %	0.69	0.78	0.57	0.62	0.63	0.64	0.64	0.65	0.78	0.57	10.15
Magnesium, %	0.31	0.29	0.31	0.35	0.32	0.33	0.30	0.32	0.35	0.29	6.30
Potassium, %	2.24	2.25	2.17	2.39	2.28	2.15	2.30	2.25	2.39	2.15	3.59
Sulfur	1163	1096	996	1160	1135	1168	1253	1160	1253	996	6.91
Micro minerals											
Copper	12.31	12.35	14.84	11.37	13.66	10.89	14.94	12.91	14.94	10.89	12.46
Iron	346.79	145.95	182.59	106.89	341.41	170.11	103.45	199.60	346.79	103.45	51.60
Manganese	50.34	35.93	33.09	26.15	29.59	24.95	29.89	32.85	50.34	24.95	26.13
Selenium	ND^a^	ND	0.13	0.08	ND	0.02	0.25	0.12	0.25	0.02	81.37
Zinc	46.99	43.79	45.65	50.03	47.80	48.76	44.83	46.84	50.03	43.79	4.74
Molybdenum	0.98	1.43	2.83	4.11	3.27	2.61	2.41	2.52	4.11	0.98	42.14
Vitamins											
Folic acid	13.54	14.26	20.77	25.02	23.10	19.39	18.85	19.28	25.02	13.54	22.02
Vitamin E	1.95	ND	2.67	2.22	2.58	2.91	ND	2.47	2.91	1.95	15.42
Thiamine	2.57	2.30	2.10	2.24	2.26	2.02	2.54	2.29	2.57	2.02	8.97

^a^ND = not detected values were below the method detection limit. Minimum detection levels for selenium and vitamin E were 0.01 mg/kg and 1 mg/kg, respectively

### Animals and experimental design

Two experiments were conducted simultaneously. A total of 16 Landrace × Yorkshire crossbred sows (parity 3) were used. Exp. 1 was designed to measure SID values for CP and AAs in seven SBM samples in non-pregnant sows. Eight animals with a T-cannula in the distal ileum were used. The T-cannula was installed according to a previous report [11]. Sows were randomly allotted to an 8 × 8 Latin square design with eight diets and eight periods. There were eight replications for each diet.

Exp. 2 was designed to measure SID values for CP and AAs in three SBM samples in mid-gestating and late-gestating sows. The three SBM sources (SBM 1, 4, and 5) were representative samples with large nutritional differences in Exp.1. Eight gestating sows were randomly allotted to a replicated 4 × 3 Youden square design, with four diets and three periods in each square in mid-gestation (d 27–47) and again in late-gestation stages (d 69–89). There were 6 replications per diet. Each period lasted 7 d, with 5 adaptation days and then 2 d collecting ileal digesta. Ileal digesta samples were collected continuously for 12 h (08:00 to 20:00 h) on d 6 and 7 in each experimental period. Specific collection methods were described in our previous research [12].

### Feeding, diets, and sample collection

Sows were individually housed in metabolism crates (2.10 m × 0.97 m × 1.20 m) with smooth sides and plastic covers. A stainless-steel feeder and nipple drinker were provided in the crates. Experiments 1 and 2 had the same dietary formulation (Table 4), and the same batch of each diet was used for all sows. The analytical values of experimental diets are shown (Table 5). An N-free diet was used to determine basal endogenous AA and CP losses. The AA digestibility of SBM was measured by a direct method with SBM as the sole N source. All diets contained 0.4% chromic oxide as an indigestible marker. Solka-Floc (International Fiber Corp., Urbana, OH, USA) was also included to increase crude fiber concentrations and prevent constipation. Water was provided ad libitum. To avoid the effects of feed intake on the SID of AA, sows were fed a daily 3 kg ration [9], with separate meals at 08:00 h and 15:00 h. Room temperature was maintained at 20 ± 2 °C. When not being tested, sows were fed standard commercial feed. Ileal digesta pretreatments before analyses were performed as described elsewhere [6].

**Table 4:** Ingredient composition (as-fed basis) in experimental diets

**Ingredient, %**	**Test diets**^**a**^	**N-free diet**
Cornstarch	44.56	76.55
Soybean meal	34.40	0.00
Soybean oil	3.00	3.00
Limestone	0.59	0.70
Dicalcium phosphate	1.90	2.20
NaCl	0.40	0.40
Sucrose	10.00	10.00
Solka floc	4.00	6.00
Chromic oxide	0.40	0.40
Choline chloride (50%)	0.25	0.25
Vitamin-mineral premix^b^	0.50	0.50
Total	100.00	100.00

^a^Test diets were formulated using different soybean meal sources

^b^Premix provided per kilogram of complete diet: 6,000 IU Vitamin A; 2,000 IU Vitamin D_3_; 80 IU Vitamin E; 3.8 mg Vitamin K; 2.0 mg Vitamin B_1_; 6.0 mg riboflavin; 4.0 mg Vitamin B_6_; 0.02 mg Vitamin B_12_; 26.0 mg niacin; 18.0 mg pantothenic acid; 3.2 mg folic acid; 0.4 mg biotin; 100 mg iron; 20 mg copper; 100 mg zinc; 25 mg manganese; 0.4 mg iodine; and 0.30 mg selenium

**Table 5:** Compositional analysis of experimental diets (% dry matter, unless otherwise indicated)

**Item**	**N-free diet**	**Test diets**^**a**^						
		**SBM 1**	**SBM 2**	**SBM 3**	**SBM 4**	**SBM 5**	**SBM 6**	**SBM 7**
Dry matter	89.79	90.44	90.29	89.67	89.85	89.73	89.96	89.26
Crude protein	0.51	15.14	15.67	16.78	17.04	16.07	15.70	16.79
Gross energy, kcal/kg	3958.71	4138.34	4179.01	4203.32	4214.49	4202.85	4207.80	4206.70
Indispensable amino acids								
Arg	0.00	1.05	1.22	1.25	1.20	1.16	1.22	1.03
His	0.00	0.41	0.45	0.45	0.43	0.42	0.47	0.39
Ile	0.01	0.61	0.69	0.77	0.76	0.70	0.80	0.66
Leu	0.02	1.09	1.25	1.34	1.30	1.27	1.38	1.14
Lys	0.01	0.95	1.07	1.07	1.01	1.00	1.04	0.89
Met	0.04	0.15	0.13	0.11	0.17	0.13	0.11	0.13
Phe	0.01	0.73	0.85	0.88	0.86	0.82	0.92	0.75
Thr	0.01	0.60	0.68	0.69	0.67	0.65	0.69	0.57
Trp	0.02	0.17	0.21	0.20	0.19	0.21	0.21	0.18
Val	0.01	0.65	0.75	0.83	0.79	0.76	0.80	0.69
Dispensable amino acids								
Ala	0.01	0.67	0.75	0.77	0.75	0.78	0.80	0.69
Asp	0.02	1.67	1.93	1.99	1.93	1.85	1.99	1.62
Cys	0.01	0.15	0.14	0.17	0.19	0.14	0.17	0.12
Glu	0.03	2.65	3.21	3.36	3.24	3.10	3.35	2.69
Gly	0.01	0.66	0.73	0.77	0.72	0.72	0.78	0.65
Pro	0.02	0.70	0.81	0.81	0.82	0.72	0.79	0.59
Ser	0.01	0.76	0.90	0.89	0.86	0.85	0.90	0.73
Tyr	0.03	0.56	0.63	0.65	0.62	0.69	0.76	0.67

^a^Soybean meal sources are described in Table 1

### Chemical analysis and calculations

Dry matter (DM), CP, crude ash, ether extract (EE), stachyose, raffinose, maltose, sucrose, AA, and mineral levels in samples were analyzed as described previously [13]. Gross energy (GE) was analyzed according to a previous report [14]. Crude fiber (CF), neutral detergent fiber (NDF), acid detergent fiber (ADF), insoluble dietary (IDF), and soluble dietary fiber (SDF) levels were also analyzed as described previously [15, 16]. Total dietary fiber (TDF) was determined as the sum of SDF and IDF. Folic acid, vitamin E, and thiamine levels were analyzed according to a previous report [17].

Nonspecific (basal) endogenous CP and AA losses were calculated from sows fed the N-free diet, and the apparent ileal digestibility (AID) and SID values of CP and AAs were calculated in test diets [18].

### Statistical analysis

The normality of residuals and outliers were tested using the UNIVARIATE procedures of SAS (SAS Institute Inc., Cary, NC, USA). Mean values deviating from treatment means by over three times the interquartile range were considered outliers and removed. Statistical data analyses were performed using PROC MIXED procedures in SAS (SAS Institute Inc.) with sow as the experimental unit. The statistical model included the fixed effect of SBM or diets and the random effects of the period, sows, and reproductive phase. When comparing differences between different pregnancy phases, a variance analysis was performed using pregnancy phase as a fixed effect and sows and periods as random effects, and only SBM 1, 4, and 5 samples were included in ileal digestibility data analyses. The LSMEANS statement was used to calculate the least squares means for each treatment, and statistical differences were separated by Tukey’s multiple range tests. Results were considered significant at *P* < 0.05 and a trend at 0.05 < *P* < 0.1.

## Results

### Chemical characteristics of SBM samples

The nutrient composition of SBM from different sources is shown in Tables 2 and 3. The average CP in samples was 50.00% (DM basis), and the coefficient of variation (CV) was 3.31%. CV values for EE, NDF, ADF, CF, SDF, TDF, carbohydrates, Trp, phosphorus, copper, iron, manganese, selenium, molybdenum, folic acid, and vitamin E were > 10%. SBM 4 had the highest CP and total AA content and lowest fiber content, whereas SBM 1 had the lowest total AA content and the highest CF, ADF, and NDF content. Maltose and manganese concentrations in SBM 1 and 2 were greater, but raffinose, selenium, molybdenum, and folic acid concentrations were lower than in other SBM. Additionally, sucrose, stachyose, and raffinose levels in SBM 4 and 7 were higher.

### Apparent ileal digestibility

All sows remained healthy during the experiments, and pregnant sows gave birth normally after the experiments. In non-pregnant sows, AID values for CP and most AAs in SBM from different origins were not different, but AID values for Ile in SBM 3 and 6 were greater when compared with SBM 1 (*P* < 0.05). The AID value for Val in SBM 2, 3, and 6 was greater than in SBM 1 (*P* < 0.05), whereas it was lower for Tyr in SBM 1, 2, 3, and 6 (Table 6). In mid-gestating sows, AID values for Arg, His, Phe, Ser, Ile, Leu, Lys, Ala, Gly, and Tyr in SBM 5 were greater than in SBM 1 (*P* < 0.05). AID values for Ile, Ala, Trp, and Asp in SBM 5 were greater than in SBM 4 (*P* < 0.05), whereas the AID value for Cys in SBM 4 and 5 was greater than in SBM 1 (*P* < 0.05) (Table 7). In late-gestating sows, the AID value for Tyr in SBM 4 and 5 was greater than in SBM 1 (*P* < 0.05), but values for other AAs were similar for SBM from different origins. When comparing sows at different gestation stages, non-pregnant sows had significantly greater AID values for Met when compared with gestating sows (*P* < 0.01), whereas non-pregnant sows had greater AID values for Trp and Ala when compared with late-gestating sows (*P* < 0.05). Also, sows had greater AID values for Lys, Val, Ala, and Asp during mid-gestation when compared with late-gestation phases (*P* < 0.05).

**Table 6:** Apparent ileal digestibility values for crude protein (CP) and amino acids (AAs) in soybean meal (SBM) from different origins in non-pregnant sows

**Item, %**	**SBM 1**	**SBM 2**	**SBM 3**	**SBM 4**	**SBM 5**	**SBM 6**	**SBM 7**	**Mean**	**SEM**	***P*-value**
CP	81.67	81.71	81.55	81.41	81.64	79.63	83.70	81.57	0.43	0.38
Indispensable AA										
Arg	90.74	92.71	91.88	90.73	91.51	92.31	91.54	91.61	0.30	0.51
His	86.74	89.28	88.01	87.46	87.60	88.47	87.87	87.87	0.30	0.49
Ile	82.18^b^	85.49^ab^	87.02^a^	83.65^ab^	85.41^ab^	87.27^a^	85.29^ab^	85.29	0.44	0.04
Leu	83.58	86.71	87.08	84.85	86.97	87.46	84.41	85.84	0.41	0.06
Lys	85.87	87.83	86.85	85.61	86.84	86.17	85.81	86.40	0.33	0.65
Met	86.60	87.90	86.84	88.84	87.99	86.35	87.99	87.52	0.35	0.44
Phe	86.23	87.51	87.19	86.86	87.64	88.31	86.56	87.18	0.35	0.77
Thr	80.80	82.93	81.97	81.30	80.79	81.91	78.94	81.25	0.48	0.50
Trp	81.55	85.55	83.75	82.35	85.73	84.94	81.76	83.64	0.50	0.09
Val	80.89^b^	85.25^a^	85.34^a^	83.34^ab^	84.02^ab^	85.16^a^	82.05^ab^	83.71	0.44	0.03
Dispensable AA										
Ala	81.68	81.17	81.55	82.53	83.18	80.75	80.75	81.69	0.47	0.82
Asp	85.37	85.98	85.70	84.31	83.76	85.77	83.59	84.91	0.47	0.70
Cys	74.13	76.32	79.94	76.83	75.26	78.53	76.03	76.73	0.64	0.23
Glu	86.72	89.30	88.52	86.90	87.71	88.41	86.41	87.69	0.41	0.48
Gly	69.78	73.39	76.43	74.98	70.57	74.75	74.05	73.42	0.88	0.37
Pro	70.98	79.61	75.71	74.46	65.35	75.55	73.37	73.66	1.51	0.29
Ser	82.80	85.40	85.35	83.83	84.46	85.23	81.69	84.11	0.46	0.23
Tyr	85.01^ cd^	84.46^d^	83.96^d^	87.12^bc^	88.71^ab^	86.15^ cd^	89.74^a^	86.48	0.41	< 0.01

^a–d^ Different superscripts indicate that means in the same row differ (*P* < 0.05)

**Table 7:** Apparent ileal digestibility values for crude protein (CP) and amino acids (AAs) in soybean meal (SBM) from different origins in sows

**Item, %**	**Non-pregnant**					**Mid gestation**					**Late gestation**					***P*-value**			
	**SBM 1-CN**	**SBM 4-BR**	**SBM 5-US**	**Mean**	**SEM**	**SBM 1-CN**	**SBM 4-BR**	**SBM 5-US**	**Mean**	**SEM**	**SBM 1-CN**	**SBM 4-BR**	**SBM 5-US**	**Mean**	**SEM**	**Non-pregnant**	**Mid gestation**	**Late gestation**	**Phase**
CP	81.67	81.41	81.64	81.57	0.82	80.29	82.62	83.35	82.06	0.67	80.00	78.85	79.65	79.45	0.70	0.99	0.13	0.80	0.06
Indispensable AA																			
Arg	90.74	90.73	91.51	91.00	0.58	90.33^b^	92.13^ab^	93.92^a^	91.89	0.52	90.07	89.66	90.98	90.15	0.48	0.83	< 0.01	0.57	0.14
His	86.74	87.46	87.60	87.27	0.60	86.56^b^	88.04^ab^	90.38^a^	88.33	0.56	87.33	86.38	88.55	87.35	0.43	0.84	< 0.01	0.11	0.34
Ile	82.18	83.65	85.41	83.74	0.90	84.80^b^	85.12^b^	88.37^a^	86.17	0.60	82.89	84.09	84.28	83.72	0.58	0.38	0.01	0.60	0.05
Leu	83.58	84.85	86.97	85.12	0.82	84.27^b^	85.04^ab^	87.61^a^	85.72	0.55	82.67	84.27	84.77	83.85	0.61	0.26	0.02	0.37	0.20
Lys	85.87	85.61	86.84	86.11^xy^	0.69	86.54^b^	87.89^ab^	89.25^a^	87.98^x^	0.43	84.47	85.15	86.16	85.15^y^	0.60	0.76	0.02	0.58	< 0.01
Met	86.60	88.84	87.99	87.85^x^	0.56	81.15	83.91	82.42	82.66^y^	0.53	83.05	82.99	81.23	82.43^y^	1.02	0.26	0.12	0.73	< 0.01
Phe	86.23	86.86	87.64	86.91	0.71	85.38^b^	86.66^ab^	88.92^a^	87.08	0.51	84.62	86.31	87.53	86.07	0.64	0.75	< 0.01	0.19	0.53
Thr	80.80	81.30	80.79	80.96	0.95	81.23	80.94	83.80	82.04	0.69	79.59	80.74	81.68	80.61	0.68	0.97	0.17	0.49	0.46
Trp	81.55	82.35	85.73	83.17^x^	1.01	80.91^ab^	79.45^b^	84.89^a^	81.75^xy^	0.92	78.18	79.57	81.45	79.74^y^	0.83	0.22	0.03	0.26	0.04
Val	80.89	83.34	84.02	82.77^xy^	0.86	83.99	84.02	86.70	84.95^x^	0.61	81.45	81.23	81.98	81.50^y^	0.64	0.32	0.10	0.91	0.01
Dispensable AA																			
Ala	81.68	82.53	83.18	82.46^x^	0.92	82.57^b^	82.55^b^	86.17^a^	83.69^x^	0.67	77.84	78.54	78.43	78.26^y^	0.80	0.83	0.03	0.93	< 0.01
Asp	85.37	84.31	83.76	84.44^xy^	0.92	84.84^ab^	83.96^b^	87.09^a^	85.32^x^	0.56	81.73	81.82	82.78	82.11^y^	0.69	0.79	0.04	0.81	0.02
Cys	74.13	76.83	75.26	75.47	1.09	71.88^b^	79.87^a^	79.16^a^	76.97	1.33	75.68	80.63	75.50	77.49	1.78	0.61	0.01	0.42	0.55
Glu	86.72	86.90	87.71	87.15	0.84	88.39	87.72	90.60	88.93	0.55	86.49	86.53	88.99	87.28	0.60	0.88	0.06	0.16	0.16
Gly	69.78	74.98	70.57	71.72	1.67	66.21^b^	73.04^ab^	77.82^a^	72.32	1.80	63.72	65.84	74.51	67.64	2.17	0.42	0.01	0.11	0.19
Pro	70.98	74.46	65.35	70.26	2.58	57.57	65.25	79.54	66.74	4.44	49.89	56.80	65.76	57.00	7.17	0.37	0.13	0.70	0.14
Ser	82.80	83.83	84.46	83.70	0.88	80.59^b^	83.96^ab^	86.23^a^	83.57	0.80	79.42	82.34	83.27	81.48	0.82	0.77	< 0.01	0.13	0.14
Tyr	85.01	87.12	88.71	87.15	0.64	85.08^b^	86.72^ab^	88.75^a^	86.96	0.54	86.23^b^	89.42^a^	89.19^a^	88.23	0.63	0.08	0.01	0.049	0.31

^a,b^Means within a row with different letters differ significantly (*P* < 0.05) in different physiological stages

^x,y^Means within a row with different lowercase superscripts indicate a significant difference between different physiological stages (*P* < 0.05)

### Endogenous AA losses in sows

The most abundant AAs in ileal endogenous protein from sows were Pro, Glu, Gly, Asp, Leu, Ser, and Thr. Basal ileal endogenous (BEL) values of indispensable AAs ranged from 80.00 mg/kg dry matter intake (DMI) for Met to 465.00 mg/kg DMI for Leu in non-pregnant sows, from 108.33 mg/kg DMI for Met to 440.00 mg/kg DMI for Leu in mid-gestating sows, and from 124.00 mg/kg DMI for Met to 505.00 mg/kg DMI for Leu in late-gestating sows (Table 8). BEL values for Met and Ala in gestating sows were greater when compared with non-pregnant sows (*P* < 0.05). The BEL value for Trp in late-gestating sows was greater than in non-pregnant sows (*P* < 0.05), the BEL value for Ile in non-pregnant sows was greater than in mid-gestating sows (*P* < 0.05), and the BEL value for Tyr in late-gestating and non-pregnant sows was greater than in mid-gestating sows (*P* < 0.05).

**Table 8:** Basal ileal endogenous losses (mg/kg dry matter intake, DMI) of crude protein (CP) and amino acid (AA) in sows^a^

**Item**	**Non-pregnant sows**	**Mid-gestating sows**	**Late-gestating sows**	**SEM**	***P*-value**
CP, g/kg DMI	9.82	10.14	10.57	0.59	0.89
Indispensable AA					
Arg	298.33	255.00	368.00	24.13	0.25
His	128.75	121.67	153.33	5.10	0.06
Ile	317.50^b^	215.00^c^	275.00^bc^	11.31	< 0.01
Leu	465.00	440.00	505.00	18.41	0.45
Lys	293.75	295.00	286.00	11.71c	0.96
Met	80.00^c^	108.33^b^	124.00^b^	4.20	< 0.01
Phe	213.75	242.00	270.00	12.48	0.23
Thr	367.50	408.00	396.67	16.54	0.62
Trp	106.25^c^	123.33^bc^	150.00^b^	5.88	0.03
Val	340.00	368.33	358.33	15.97	0.78
Dispensable AA					
Ala	340.00^c^	468.00^b^	456.00^b^	19.65	0.04
Asp	575.00	658.00	628.00	25.55	0.44
Cys	171.25	132.50	135.00	8.77	0.17
Glu	692.50	810.00	712.00	34.31	0.45
Gly	674.29	741.67	906.00	69.33	0.46
Pro	1920.00	1800.00	2556.00	314.03	0.65
Ser	358.75	436.00	392.00	19.21	0.32
Tyr	182.86^b^	30.00^c^	211.67^b^	7.69	< 0.01

^a^Each mean represents eight and six observations for non-pregnant and pregnant sows, respectively

^b,c^Different superscripts indicate that means in the same row differ (*P* < 0.05)

### Standardized ileal digestibility

As indicated (Table 9), for non-pregnant sows, the SID of CP and most AAs in SBM from different sources were not different, except the SID of Tyr in SBM 5 and 7 were greater than that in SBM 1, 2, 3, and 6 (*P* < 0.05). The average AA SID values for Pro and Arg were the highest (102.58% and 94.21%), whereas those for Gly, Ala, and Thr values were the lowest (84.17%, 86.26%, and 86.95%, respectively). In non-pregnant and late-gestating sows, no differences were observed in the SID of CP and all AA between SBM 1, 4, and 5 (Table 10). In mid-gestating sows, the SID values of Arg, His, Lys, Phe, Gly, Ser, and Tyr in SBM 5 were greater than in SBM 1 (*P* < 0.05), SID values for Ile, Ala, and Asp in SBM 5 were greater than in SBM 4 (*P* < 0.05), the SID value of Leu in SBM 5 was greater than in SBM 1 and 4 (*P* < 0.05), and SID values of Cys in SBM 4 and 5 were significantly greater than in SBM 1 (*P* < 0.01) (Table 10).

**Table 9:** Standardized ileal digestibility values for crude protein (CP) and amino acids (AAs) in soybean meal (SBM) from different origins in non-pregnant sows

**Item, %**	**SBM 1**	**SBM 2**	**SBM 3**	**SBM 4**	**SBM 5**	**SBM 6**	**SBM 7**	**Mean**	**SEM**	***P*-value**
CP	88.16	87.98	87.40	87.17	87.75	85.88	89.55	87.64	0.43	0.45
Indispensable AA										
Arg	93.60	95.17	94.28	93.23	94.10	94.77	94.45	94.21	0.29	0.63
His	89.91	92.17	90.90	90.48	90.70	91.24	91.20	90.90	0.30	0.64
Ile	87.43	90.13	91.18	87.86	89.98	91.27	90.14	89.67	0.45	0.13
Leu	87.89	90.47	90.59	88.47	90.67	90.87	88.53	89.61	0.40	0.18
Lys	88.92	90.54	89.56	88.48	89.74	88.96	89.07	89.30	0.33	0.79
Met	91.27	93.28	93.20	92.96	93.37	92.71	93.37	92.87	0.35	0.70
Phe	89.11	89.98	89.58	89.30	90.20	90.59	89.36	89.72	0.34	0.92
Thr	86.97	88.37	87.33	86.82	86.48	87.27	85.43	86.95	0.46	0.84
Trp	88.02	90.79	89.25	88.14	90.97	90.18	87.87	89.29	0.48	0.37
Val	86.12	89.78	89.44	87.64	88.49	89.41	86.98	88.24	0.42	0.15
Dispensable AA										
Ala	86.75	85.70	85.97	87.06	87.54	85.00	85.68	86.26	0.47	0.82
Asp	88.84	88.99	88.61	87.32	86.90	88.68	87.17	88.05	0.46	0.80
Cys	85.46	88.46	89.94	86.82	87.40	88.53	90.20	88.11	0.62	0.37
Glu	89.32	91.45	90.57	89.03	89.94	90.47	88.98	89.95	0.40	0.68
Gly	81.45	83.94	86.43	85.67	81.26	84.62	85.90	84.17	0.87	0.54
Pro	103.12	107.39	103.49	101.90	89.43	104.03	111.51	102.58	1.95	0.09
Ser	87.54	89.40	89.39	88.02	88.70	89.23	86.62	88.41	0.45	0.58
Tyr	88.58^bc^	87.63^c^	87.04^c^	90.35^ab^	91.61^a^	88.78^bc^	92.73^a^	89.55	0.40	< 0.01

^a–c^Different superscripts indicate that means in the same row differ (*P* < 0.05)

**Table 10:** Standardized ileal digestibility values for crude protein (CP) and amino acids (AAs) in soybean meal (SBM) from different origins in sows

**Item, %**	**Non-pregnant**					**Mid gestation**					**Late gestation**					***P*-value**			
	**SBM 1-CN**	**SBM 4-BR**	**SBM 5-US**	**Mean**	**SEM**	**SBM 1-CN**	**SBM 4-BR**	**SBM 5-US**	**Mean**	**SEM**	**SBM 1-CN**	**SBM 4-BR**	**SBM 5-US**	**Mean**	**SEM**	**Non-pregnant**	**Mid gestation**	**Late gestation**	**Phase**
CP	88.16	87.17	87.75	87.67	0.82	86.99	88.57	89.66	88.40	0.64	86.98	85.17	86.28	86.07	0.72	0.89	0.22	0.59	0.13
Indispensable AA																			
Arg	93.60	93.23	94.10	93.64	0.58	92.78^b^	94.21^ab^	96.03^a^	94.12	0.49	94.50	93.45	94.83	94.17	0.48	0.83	0.02	0.49	0.74
His	89.91	90.48	90.70	90.37	0.60	89.56^b^	90.71^ab^	93.05^a^	91.11	0.54	90.04	89.79	91.96	90.52	0.54	0.88	0.02	0.23	0.63
Ile	87.43	87.86	89.98	88.40	0.89	88.29^ab^	88.13^b^	91.34^a^	89.31	0.58	87.33	87.93	88.12	87.77	0.57	0.49	0.02	0.85	0.38
Leu	87.89	88.47	90.67	88.98	0.80	88.31^b^	88.37^b^	90.99^a^	89.28	0.53	87.31	88.13	88.69	88.01	0.59	0.36	0.04	0.66	0.43
Lys	88.92	88.48	89.74	89.05^xy^	0.69	89.73^b^	90.69^ab^	92.08^a^	90.91^x^	0.41	87.56	87.86	88.92	88.01^y^	0.59	0.77	0.04	0.69	< 0.01
Met	91.27	92.96	93.37	92.55^x^	0.55	87.62	90.38	88.89	89.13^y^	0.53	90.11	90.05	88.29	89.49^y^	1.02	0.28	0.12	0.73	< 0.01
Phe	89.11	89.30	90.20	89.53	0.71	88.71^b^	89.33^ab^	91.71^a^	89.99	0.48	88.37	89.31	90.71	89.39	0.61	0.82	0.02	0.33	0.80
Thr	86.97	86.82	86.48	86.76	0.95	88.06	86.80	89.83	88.24	0.69	86.26	86.45	87.56	86.71	0.66	0.98	0.18	0.73	0.34
Trp	88.02	88.14	90.97	89.00	0.98	87.97	86.12	90.60	88.23	0.86	87.00	87.90	88.59	87.83	0.77	0.41	0.09	0.71	0.63
Val	86.12	87.64	88.49	87.42^y^	0.83	89.28	88.76	91.57	89.90^x^	0.60	86.59	85.85	86.78	86.36^y^	0.64	0.54	0.11	0.84	< 0.01
Dispensable AA																			
Ala	86.75	87.06	87.54	87.11^xy^	0.91	89.29^ab^	88.55^b^	92.09^a^	89.89^x^	0.64	84.71	84.76	84.56	84.68^y^	0.80	0.95	0.05	1	< 0.01
Asp	88.84	87.32	86.90	87.64^xy^	0.92	88.86^ab^	87.26^b^	90.53^a^	88.89^x^	0.56	85.57	84.97	86.10	85.55^y^	0.71	0.70	0.04	0.82	0.02
Cys	85.46	86.82	87.40	86.56	1.07	79.88^b^	90.21^a^	93.15^a^	87.75	1.61	85.01	88.41	83.35	85.72	1.67	0.77	< 0.01	0.49	0.62
Glu	89.32	89.03	89.94	89.47	0.84	91.45	90.21	93.15	91.61	0.55	89.11	88.64	91.19	89.55	0.56	0.91	0.07	0.16	0.06
Gly	81.45	85.67	81.26	82.72	1.65	77.59^b^	83.32^ab^	87.69^a^	82.84	1.70	77.72	78.48	86.81	80.66	2.11	0.50	0.03	0.17	0.65
Pro	103.12	101.90	96.60	100.54	2.52	83.66	86.43	101.49	89.88	4.29	86.99	87.28	96.98	90.03	7.08	0.56	0.22	0.84	0.18
Ser	87.54	88.02	88.70	88.08	0.88	86.54^b^	88.90^ab^	91.23^a^	88.89	0.72	84.69	86.72	87.75	86.21	0.77	0.88	0.01	0.28	0.09
Tyr	88.58	90.35	91.61	90.35^x^	0.61	85.63^b^	87.18^ab^	89.21^a^	87.44^y^	0.53	90.05	92.70	92.47	91.70^x^	0.59	0.17	0.01	0.11	< 0.01

^a,b^Means within a row with different letters differ significantly (*P* < 0.05) in different physiological stages

^x,y^Means within a row with different lowercase superscripts indicate a significant difference between different physiological stages (*P* < 0.05)

When comparing sows at different gestation stages, sows in mid-gestation had greater SID values of Lys, Ala, and Asp when compared with those in late-gestation stages (*P* < 0.05), whereas non-pregnant sows had significantly greater SID value of Met than gestating sows (*P* < 0.01). Mid-gestating sows had greater SID value of Val and lower SID value for Tyr than non-pregnant and late-gestating sows (*P* < 0.01).

## Discussion

### Chemical characteristics of SBM samples

In the SBM samples, levels of CP, EE, CF, NDF, ADF, Ash, and AA were within previously reported range values [9, 16, 19]. IDF, SDF, and TDF values in SBM concurred with previous data, with no difference in values from different countries [20]. SBM 4 was dehulled, which may have accounted for lower IDF, SDF, and TDF levels. SBM 1 and 2 had higher and lower maltose and raffinose concentrations, respectively, which were possibly due to different growing conditions and soybean varieties [21]. As previously reported, commercial SBM produced from soybeans in China had lower AA concentrations and higher ADF and NDF concentrations [6], which may be due to Chinese SBM containing more soybean hulls. SBM 1 had the lowest Lys to CP ratio, indicating possible overprocessing or heat damage [22]. Thus, soybean origin, growth, and agronomic conditions substantially affect the chemical composition of SBM [5].

### Apparent ileal digestibility

To the best of our knowledge, few articles have reported AID values for CP and AAs of SBM in gestating sows [23]. AID values for His, Ile, Leu, Lys, Met, Phe, Trp, Val, Asp, Cys, Glu, Ser, and Tyr in SBM in non-pregnant sows concurred with previous reports, whereas AID values for CP and other AAs were higher than reported previously [23]. In addition, AID values for CP, Thr, Val, and Ala in SBM in mid-gestating sows were higher, Met AID values were slightly lower, and AID values for other AAs were similar to those of Stein et al. [23]. AID values for CP in SBM in late-gestating sows were higher, AID values for Met, Lys, Trp, and Asp were lower, and AID values for other AAs were similar to those of Stein et al. [23]. However, Stein et al. did not fully describe sow gestation periods in their studies [23].

In our study, apart from the AID values for Trp, Cys, Ile, and Pro in non-pregnant sows, which were similar, the mean AID values for other AAs were higher than mean AID values of solvent-extracted SBM published in NRC (2012) [9]. In observed differences, the highest value was for Val (4.71%) and the lowest value was for Lys (1.40%). In mid-gestating sows, the mean AID values of most AAs were higher than the values of solvent-extracted SBM published by the NRC (2012) [9], with the largest difference being Val (5.95%) and the lowest difference being Arg (1.89%). Additionally, the mean AID value for Met was lower (2.34%), whereas AID values for Trp, Cys, and Pro were similar. In late-gestating sows, the mean AID values for His, Thr, Val, Glu, and Tyr were higher than published solvent-extracted SBM values from the NRC (2012) [9], with the largest difference being Tyr (5.23%) and the lowest difference being Glu (1.28%). Additionally, mean AID values for Met, Trp, Ala, Asp, and Pro were lower when compared with the NRC (2012) [9], with the largest difference being Pro (17%) and the lowest difference being Ala (0.74%). The NRC (2012) data came from growth-finishing pigs, which may explain these discrepancies [3].

In SBM 1, AID values for Ile, Val, and Tyr were lower during non-gestation periods, AID values were lower for Arg, His, Ile, Leu, Lys, Phe, Cys, Gly, Ser, and Tyr during mid-gestation periods, and lower for Tyr during late-gestation periods. These observations were possibly due to greater CF, NDF, and ADF concentrations in SBM 1. It was reported that NDF negatively affected AID values for CP and AAs in pigs [24, 25]. Soy hulls may reduce AA digestibility [25]. In our study, the AIDs of Lys, Met, Trp, Val, Ala, and Asp of SBM were different at different sow gestation stages. However, few studies have examined the mechanisms by which sow gestation stage affects nutrient digestion. This will be examined in future research.

### Endogenous AA losses in sows

To meet the nutritional needs of pigs, SID values for CP and AAs are important reference indices for diet formulations. The approach requires the accurate determination of ileal endogenous AA losses in pigs, with N-free diets traditionally used to calculate SID values [26, 27]. Ileal endogenous amino acid (IEAA) losses are considered inevitable losses [28]. The highest endogenous amino acid loss was Pro, regardless of sow gestation status. This observation is consistent with a previous report [28]. In our study, basal endogenous CP and AA losses in sows were lower than previously reported, where a casein-cornstarch diet was used [10]. This is consistent with a previous report showing that animals fed a casein diet had higher IEAA losses than those fed an N-free diet [29]. Stein et al. measured IEAA losses in restricted-fed gestating sows using an N-free diet, with slightly higher results than ours [30]. This difference may be due to differences in tested sows and different ingredient composition in N-free diets [26]. In growing animals, basal endogenous CP loss is reportedly closely related to DM intake and BW [31]. It was reported that IEAAs in growing pigs tend to decrease with increasing BW [32]. From our experimental results, under similar feed intake conditions, different pregnancy states may have affected the basal endogenous losses of Ile, Met, Trp, Ala, and Tyr.

### Standardized ileal digestibility

Currently, the SID values for AAs of SBM in sows are scarce, which impacts the precise use of SBM in sow feed. In this study, the SID values for CP and AAs of SBM were lower than previously reported for SBM values in pregnant sows, regardless of their gestation status [8]. This was possibly due to differences in test subjects and methods. Additionally, apart from similar SID values for Trp, Ala, and Ser in non-pregnant sows, mean SID values for other AAs were higher when compared with solvent-extracted SBM values from the NRC (2012) [9]. Among these differences, the largest difference was His (4.90%) and the lowest was Gly (1.17%). The mean SID values for His and Gly of the 7 SBM samples (90.90% and 84.17%) were higher than the values (86.00% and 83.00%) published by NRC [9]. For mid-gestating sows, apart from similar SID values for Met, Trp, Gly, Pro, and Ser, mean SID values for other AAs were higher when compared with NRC data (2012)—the largest difference was Val (5.90%) and the lowest was Ile (1.31%). The mean SID values for Val and Ile of the 3 SBM samples (89.90% and 89.31%) were higher than the values (84.00% and 88.00%) published by NRC [9]. These observations were consistent with our previous findings showing that SBM had higher effective energy values in sows when compared with growing pigs [3]. For late-gestating sows, mean SID values for Arg, His, Leu, Phe, Thr, Val, Glu, and Tyr were higher when compared with NRC data (2012)—the largest difference was Tyr (5.70%) and the lowest was Glu (1.55%). The mean SID values for Tyr and Glu of the 3 SBM samples (91.70% and 89.55%) were higher than the values (86.00% and 88.00%) published by NRC [9]. Additionally, mean SID values for Trp, Ala, Pro, and Ser were lower than NRC data (2012)—the largest difference was Pro (7.97%) and the lowest was Ala (1.32%). The mean SID values for Pro and Ala of the 3 SBM samples (90.03% and 84.68%) were lower than the values (98.00% and 86.00%) published by NRC [9]. We believe our study data will contribute to the accurate and efficient use of SBM in sow diets. It is important to note that since the requirements for SID AAs varied greatly through gestation [9], our study data will be more valuable when pig producers start using multi-stage diets in gestation and practice precision feeding.

Higher fiber content in ingredients may prevent proteases from binding to proteins, resulting in lower AA digestibility in growing pigs [33, 34]. This may also increase digesta flow, shorten digestion times, and increase exogenous and endogenous protein loss [35, 36]. In our study, this may partially explain standard ileal AA digestibility differences between SBM samples. SBM 1 had the highest CF, NDF, and ADF levels and relatively lower AA digestibility, its raw material (soybeans) came from China. We observed that the SID values for Lys, Met, Val, Ala, Asp, and Tyr in SBM were different at different sow gestation stages. These observations suggest that pregnancy stages affect the SID values of AA in SBM. However, few studies have explained this phenomenon, and specific reasons require further study. Some differences in the SID values of AA between different SBM samples were observed only at mid-gestation stage. This may be due to stronger metabolic activity and higher alpha diversity values of gut microorganisms in non-gestation and late-gestation stages, and also higher fiber digestibility, resulting in weakened fiber effects on AA digestibility [37, 38].

## Conclusions

In summary, we revealed the SID of AAs in SBM from different sources when fed to non-pregnant and pregnant sows, and SID values of AAs can be affected by CF, NDF, and ADF content in SBMs fed to mid-gestating sows. In addition, the SID values for Lys, Met, Val, Ala, Asp, and Tyr in SBM were affected by sow gestation stages. Our findings provide a comprehensive reference point for accurate SBM use in the diet of sows.

## Data Availability

All data generated or analyzed during this study are available from the corresponding author upon reasonable request.

## Abbreviations

AA: Amino acid
ADF: Acid detergent fiber
AID: Apparent ileal digestibility
BEL: Basal ileal endogenous
BW: Body weight
CF: Crude fiber
CP: Crude protein
CV: Coefficient of variation
DM: Dry matter
DMI: Dry matter intake
EE: Ether extract
GE: Gross energy
IDF: Insoluble dietary fiber
IEAAs: Ileal endogenous amino acids
NDF: Neutral detergent fiber
N: Nitrogen
SBM: Soybean meal
SDF: Soluble dietary fiber
SID: Standardized ileal digestibility
TDF: Total dietary fiber

## References

[1] Noblet J, Wu S-B, Choct M (2022). Methodologies for energy evaluation of pig and poultry feeds: a review. Anim Nut.

[2] Yan H, Jin JQ, Yang P, Yu B, He J, Mao XB (2022). Fermented soybean meal increases nutrient digestibility via the improvement of intestinal function, anti-oxidative capacity and immune function of weaned pigs. Animal.

[3] Wang K, Zou X, Guo L, Huang L, Wang Y, Yang P (2022). The nutritive value of soybean meal from different sources for sows during mid- and late gestation. J Anim Sci..

[4] Sotak-Peper KM, Gonzalez-Vega JC, Stein HH (2015). Concentrations of digestible, metabolizable, and net energy in soybean meal produced in different areas of the United States and fed to pigs. J Anim Sci.

[5] García-Rebollar P, Cámara L, Lázaro RP, Dapoza C, Pérez-Maldonado R, Mateos GG (2016). Influence of the origin of the beans on the chemical composition and nutritive value of commercial soybean meals. Anim Feed Sci Technol.

[6] Lagos LV, Stein HH (2017). Chemical composition and amino acid digestibility of soybean meal produced in the United States, China, Argentina, Brazil, or India. J Anim Sci.

[7] Pedersen C, Almeida JS, Stein HH (2016). Analysis of published data for standardized ileal digestibility of protein and amino acids in soy proteins fed to pigs. J Anim Sci.

[8] Stein HH, Kim SW, Nielsen TT, Easter RA (2001). Standardized ileal protein and amino acid digestibility by growing pigs and sows. J Anim Sci.

[9] NRC (2012). Nutrient Requirements of Swine.

[10] Velayudhan DE, Hossain MM, Stein HH, Nyachoti CM (2019). Standardized ileal digestibility of amino acids in canola meal fed to gestating and lactating sows. J Anim Sci.

[11] Stein HH, Shipley CF, Easter RA (1998). Technical note: A technique for inserting a T-cannula into the distal ileum of pregnant sows. J Anim Sci.

[12] Wang Y, Ma X, Li G, Sun M, Xu S, Lin Y, et al. Effects of feeding levels on ileal amino acid digestibility of extruded full fat soybeans in non-gestating sows. J Anim Sci. 2023;kad052. 10.1093/jas/skad052. 10.1093/jas/skad052 PMC10681036 36807524

[13] AOAC International. Official Methods of Analysis of AOAC International. 18th ed. Rev. 2nd ed. In: Hortwitz W, Latimer Jr GW, editors. Gaithersburg: AOAC International; 2007.

[14] Wang L, Zhou J, Chen Y, Wang L, Pan H, Hu Q (2021). Chemical composition, energy content, and amino acid digestibility in Cyperus esculentus co-products fed to growing pigs. J Anim Sci.

[15] Navarro DMDL, Bruininx EMAM, de Jong L, Stein HH (2018). The contribution of digestible and metabolizable energy from high-fiber dietary ingredients is not affected by inclusion rate in mixed diets fed to growing pigs. J Anim Sci.

[16] Li Z, Wang X, Guo P, Liu L, Piao X, Stein HH (2015). Prediction of digestible and metabolisable energy in soybean meals produced from soybeans of different origins fed to growing pigs. Arch Anim Nutr.

[17] Cobas N, Pineiro-Lago L, Gomez-Limia L, Franco I, Martinez S (2021). Vitamin retention during the canning of swordfish (Xiphias gladius) with different filling media. J Food Sci.

[18] Stein HH, Seve B, Fuller MF, Moughan PJ, de Lange CF, Committee on Terminology to Report AA Bioavailability and Digestibility (2007). Invited review: amino acid bioavailability and digestibility in pig feed ingredients: terminology and application. J Anim Sci..

[19] Ibáñez MA, de Blas C, Cámara L, Mateos GG (2020). Chemical composition, protein quality and nutritive value of commercial soybean meals produced from beans from different countries: a meta-analytical study. Anim Feed Sci Technol.

[20] Lopez DA, Lagos LV, Stein HH (2020). Digestible and metabolizable energy in soybean meal sourced from different countries and fed to pigs. Anim Feed Sci Technol.

[21] Hollung K, Øverland M, Hrustić M, Sekulić P, Miladinović J, Martens H (2005). Evaluation of nonstarch polysaccharides and oligosaccharide content of different soybean varieties (Glycine max) by near-infrared spectroscopy and proteomics. J Agric Food Chem.

[22] González-Vega JC, Kim BG, Htoo JK, Lemme A, Stein HH (2011). Amino acid digestibility in heated soybean meal fed to growing pigs. J Anim Sci.

[23] Stein HH, Aref S, Easter RA (1999). Comparative protein and amino acid digestibilities in growing pigs and sows. J Anim Sci.

[24] Sulabo RC, Ju WS, Stein HH (2013). Amino acid digestibility and concentration of digestible and metabolizable energy in copra meal, palm kernel expellers, and palm kernel meal fed to growing pigs. J Anim Sci.

[25] Dilger RN, Sands JS, Ragland D, Adeola O (2004). Digestibility of nitrogen and amino acids in soybean meal with added soyhulls. J Anim Sci.

[26] Zhou H, Wu W, Mahmood T, Chen Y, Xu Y, Wang Y (2022). Comparison of endogenous amino acid losses in broilers when offered nitrogen-free diets with differing ratios of dextrose to corn starch. Sci Rep.

[27] Park CS, Ragland D, Helmbrecht A, Htoo JK, Adeola O (2019). Digestibility of amino acid in full-fat canola seeds, canola meal, and canola expellers fed to broiler chickens and pigs. J Anim Sci.

[28] Jansman AJM, Smink W, van Leeuwen P, Rademacher M (2002). Evaluation through literature data of the amount and amino acid composition of basal endogenous crude protein at the terminal ileum of pigs. Anim Feed Sci Technol.

[29] Adeola O, Xue PC, Cowieson AJ, Ajuwon KM (2016). Basal endogenous losses of amino acids in protein nutrition research for swine and poultry. Anim Feed Sci Technol.

[30] Stein HH, Trottier NL, Bellaver C, Easter RA (1999). The effect of feeding level and physiological status on total flow and amino acid composition of endogenous protein at the distal ileum in swine. J Anim Sci.

[31] Moughan PJ, Rutherfurd SM (2012). Gut luminal endogenous protein: Implications for the determination of ileal amino acid digestibility in humans. Br J Nutr.

[32] Pahm AA, Pedersen C, Hoehler D, Stein HH (2008). Factors affecting the variability in ileal amino acid digestibility in corn distillers dried grains with solubles fed to growing pigs. J Anim Sci.

[33] Wang L, Zeng Z, Hu Q, Wang L, Shi H, Lai C, et al. Determination and prediction of the available energy and amino acids digestibility of full-fat soybean fed to growing pigs. J Anim Sci. 2023;101:skac395. 10.1093/jas/skac395. 10.1093/jas/skac395 PMC9985155 36444860

[34] Lenis NP, Bikker P, van der Meulen J, van Diepen JT, Bakker JGM, Jongbloed AW (1996). Effect of dietary neutral detergent fiber on ileal digestibility and portal flux of nitrogen and amino acids and on nitrogen utilization in growing pigs. J Anim Sci.

[35] Sauer WC, Ozimek OL (1986). Digestibility of amino acids in swine: Results and their practical applications. Livest Prod Sci.

[36] Schulze H, van Leeuwen P, Verstegen MWA, Huisman J, Souffrant WB, Ahrens F (1994). Effect of level of dietary neutral detergent fiber on ileal apparent digestibility and ileal nitrogen losses in pigs. J Anim Sci.

[37] Zhuo Y, Feng B, Xuan Y, Che L, Fang Z, Lin Y (2020). Inclusion of purified dietary fiber during gestation improved the reproductive performance of sows. J Anim Sci Biotechnol.

[38] Ji Y, Li H, Xie P, Li Z, Li H, Yin Y (2019). Stages of pregnancy and weaning influence the gut microbiota diversity and function in sows. J Appl Microbiol.

